# *Salmonella* Typhimurium Invalidated for the Three Currently Known Invasion Factors Keeps Its Ability to Invade Several Cell Models

**DOI:** 10.3389/fcimb.2018.00273

**Published:** 2018-08-10

**Authors:** Sylvie M. Roche, Sébastien Holbert, Jérôme Trotereau, Samantha Schaeffer, Sonia Georgeault, Isabelle Virlogeux-Payant, Philippe Velge

**Affiliations:** ^1^ISP, Institut National de la Recherche Agronomique (INRA), UMR 1282, Université de Tours, Paris, France; ^2^INSERM UMR 1162, Institut de Génétique Moléculaire, Paris, France; ^3^Plateforme des Microscopies, Université et CHRU de Tours, Tours, France

**Keywords:** T3SS-1, T3SS-1 independent invasion, zipper, trigger, entry mechanism, cell models

## Abstract

To establish an infection, *Salmonella* has to interact with eukaryotic cells. Invasion of non-phagocytic cells (i.e., epithelial, fibroblast and endothelial cells) involves either a trigger or a zipper mechanism mediated by the T3SS-1 or the invasin Rck, respectively. Another outer membrane protein, PagN, was also implicated in the invasion. However, other unknown invasion factors have been previously suggested. Our goal was to evaluate the invasion capability of a *Salmonella* Typhimurium strain invalidated for the three known invasion factors. Non-phagocytic cell lines of several animal origins were tested in a gentamicin protection assay. In most cells, we observed a drastic decrease in the invasion rate between the wild-type and the triple mutant. However, in five cell lines, the triple mutant invaded cells at a similarly high level to the wild-type, suggesting the existence of unidentified invasion factors. For the wild-type and the triple mutant, scanning-electron microscopy, confocal imaging and use of biochemical inhibitors confirmed their cellular uptake and showed a zipper-like mechanism of internalization involving both clathrin- and non-clathrin-dependent pathways. Despite a functional T3SS-1, the wild-type bacteria seemed to use the same entry route as the mutant in our cell model. All together, these results demonstrate the existence of unknown *Salmonella* invasion factors, which require further characterization.

## Introduction

*Salmonella enterica* serovar Typhimurium (*S*. Typhimurium) is one of the broad host range serotypes incriminated in food-borne diseases in industrial countries. In the European Union, over 90,000 salmonellosis cases are reported every year (EFSA., 2017) and between 2008 and 2013 in France, *Salmonella* spp ranked as the third cause of foodborne illnesses (12%), as the second cause of hospitalization (24%), and as the first cause of death (27%) (Van Cauteren et al., 2017). The bacteria are commonly found in the intestinal tracts of healthy birds and mammals, resulting in a spectrum of outcomes ranging from severe systemic disease to asymptomatic carriage (Velge et al., 2012). In calves, the Typhimurium serovar causes enterocolitis, and infected animals can succumb to dehydration. In newly hatched chicks, it causes systemic disease and diarrhea, whereas older chickens are asymptomatic carriers. It could also be responsible for a typhoid fever like disease in susceptible mouse strains (Santos et al., 2001).

*Salmonella* is a facultative intracellular bacterium/pathogen able to interact with and to invade non-phagocytic eukaryotic cells both *in vitro* and *in vivo* (Finlay and Brumell, 2000; De Jong et al., 2012). Invasion of these cells is considered as one of the most important steps of *Salmonella* pathogenesis. The most extensively investigated invasion mechanism requires the Type III Secretion System-1 (T3SS-1) encoded by the *Salmonella* pathogenicity island 1 (SPI-1), a needle-like structure which directly injects bacterial effector proteins into the host cell cytoplasm to manipulate cell signaling pathways leading to actin cytoskeletal rearrangement and bacterial internalization (Ly and Casanova, 2007). The T3SS-1 mediates invasion by a trigger mechanism, corresponding to intense membrane ruffling which envelops the bacterium, and leads to its internalization (Francis et al., 1992). Other entry mechanisms involving Rck and PagN, two outer membrane proteins, have been described in *Salmonella* (Heffernan et al., 1994; Heithoff et al., 1999; Lambert and Smith, 2008).

Rck is poorly expressed *in vitro* under standard culture conditions, but its expression is induced by quorum-sensing and controlled through the quorum-sensing transcriptional regulator SdiA (Abed et al., 2014). The epidermal growth factor receptor has been identified as the cell signaling receptor required for Rck-mediated adhesion and internalization (Wiedemann et al., 2016). Rck invasion induces a local accumulation of actin, leading to discrete membrane rearrangements, characteristic of a zipper entry process (Rosselin et al., 2010). The second outer membrane protein, PagN is another invasin, whose expression is regulated by the two-component regulatory system PhoP–PhoQ. Acidic pH and a low Mg^2+^ concentration are required for its optimal *in vitro* expression (Lambert and Smith, 2008). PagN of *S*. Typhimurium utilizes heparinated proteoglycans to invade mammalian cells (Lambert and Smith, 2009). *Salmonella* is therefore the first bacterium known to be able to induce both zipper (Rosselin et al., 2010) and trigger mechanisms to invade host cells.

For a long time, T3SS-1 was considered as the only invasion factor. However, several studies have shown that a SPI-1 or a *invA* mutant remains invasive and pathogenic *in vivo* (Murray and Lee, 2000; Hapfelmeier et al., 2005; Desin et al., 2009) and *in vitro* (Aiastui et al., 2010; Radtke et al., 2010; Van Sorge et al., 2011). Moreover, a T3SS-1 mutant cultivated in conditions which do not allow the expression of Rck and PagN keeps its ability to invade some cells (Rosselin et al., 2011). Although clear evidence is lacking, all these papers tend to suggest the existence of unknown entry routes.

The cellular internalization of exogenous particles is a physiological process and distinct internalization pathways have been identified in mammalian cells. Endocytosis is a well-documented phenomenon (Le Roy and Wrana, 2005; Sigismund et al., 2012). An example is macropinocytosis, a receptor-independent endocytic pathway, which is associated with actin-dependent plasma membrane ruffling (Maréchal et al., 2001; Hänisch et al., 2012). In clathrin-mediated endocytosis, transmembrane receptors bind with their ligands and are clustered into clathrin-coated pits (Mcmahon and Boucrot, 2011) resulting in the formation of vesicles, which are either recycled to the surface membrane or fuse with lysosomes. Another pathway is clathrin-independent but lipid-raft dependent that includes caveolae, which are small vesicles enriched with caveolin, cholesterol and sphingolipids (Parton and Richards, 2003; Le Roy and Wrana, 2005). These endocytic entry processes are used by numerous bacteria and viruses to invade cells (Cossart and Helenius, 2014). As multiple endocytic pathways exist in a single cell, the development of specific inhibitors has helped in identifying the molecules involved in the cross-talk between these pathways (Mayor et al., 2014).

The aim of our study was to identify the invasion capabilities of the *S*. Typhimurium 14028 strain and its isogenic triple mutant invalidated for its T3SS-1 (Δ*invA*) and deleted for the genes encoding the invasion factors Rck and PagN (Δ*invA::kan* Δ*pagN::cm* Δ*rck*) (STM-3Δ). Different non-phagocytic cell lines and primary cells from several animal origins, representing cell types encountered by *Salmonella* within their hosts were used to evaluate the relative importance of the known invasion factors.

## Materials and methods

### Bacterial strains and plasmids

The different bacteria and plasmids used are described in Table 1. The bacterial strains were maintained in TSB (Tryptic Soy Broth–Difco)-glycerol (25%) at −80°C. For the invasion assays, bacteria were first subcultured in TSB at 37°C for 7–8 h with shaking in the presence of carbenicillin (100 μg/mL), kanamycin (50 μg/mL), chloramphenicol (30 μg/mL) and nalidixic acid (100 μg/mL) when required. The bacteria were then grown overnight in TSB without shaking and bacteria concentrations were standardized turbidimetrically and diluted appropriately in cell culture medium.

**Table 1:** Strains, plasmids and primers used in this study.

**Strains**	**Designation used in the article**	**References**
*Salmonella* Typhimurium 14028	WT	ATCC
*Salmonella* Typhimurium 14028 Δ*invA*::*kan* Δ*pagN*::*cm* Δ*rck*	STM-3Δ	This study
*Salmonella* Typhimurium 14028 Δ*invA*::*kan*	STM-Δ*invA*	This study
*Escherichia coli* MC1061	*E. coli* MC1061	Casadaban and Cohen, 1980
**Plasmids**	**Characteristics**	
pGG2-DsRed	Plasmid carrying DsRed (Cb^R^)	Lelouard et al., 2010
pCX340	Plasmid carrying *β-lactamase* gene (Tc^R^)	Fookes et al., 2011
pCX340sopD	Plasmid carrying sopD *β-lactamase* fusion (Tc^R^)	Fookes et al., 2011
pKD3	Vector carrying an FRT-Cm-FRT cassette (Cb^R^ Cm^R^)	Datsenko and Wanner, 2000
pKD4	Vector carrying an FRT-Kan-FRT cassette (Cb^R^ Kan^R^)	Datsenko and Wanner, 2000
pK46	Carries genes encoding λ-Red recombinase, Temperature sensitive replication (Cb^R^)	Datsenko and Wanner, 2000
pCP20	Carries genes encoding FLP recombinase, Temperature sensitive replication (Cb^R^)	Cherepanov and Wackernagel, 1995
**Primers**	**Sequence**	
rck-P1	5′-ATCATGAAAAAAATCGTTCTGTCCTCACTGCTGCTGTCCGCAGCCGGGCTG TGTAGGCTGGAGCTGCTTC-3′	This study
rck-P2	5′-CTCCGCTCCCTTTCCTGCTCTCCGTTATCAGAACCGGTAACCGACACCAAC ATATGAATATCCTCCTTAG-3′	This study
P1-pagN	5′-GAAACTTGTCTTTTAGCCCAATATTAAGGCAGGTTCTGAAATGAAAAACTG TGTAGGCTGGAGCTGCTTC-3′	This study
P2-pagN	5′-CCTTCGGGAACCCACAGGACCAGCTATTTTACCGATAGTGTTTAAAAGGCCA TATGAATATCCTCCTTAG-3′	This study
P1-invA-TAA	5′-TTATATTGTTTTTATAACATTCACTGACTTGCTATCTGCTATCTCACCGAGTG TAGGCTGGAGCTGCTTC-3′	This study
P2-invA-GTG	5′-GTGCTGCTTTCTCTACTTAACAGTGCTCGTTTACGACCTGAATTACTGATCATA TGAATATCCTCCTTAG-3′	This study

### Construction of the different mutants

Deletion of the *invA, rck* and *pagN* open reading frames was performed using the λ Red recombinase method (Datsenko and Wanner, 2000) using primers described in Table 1. Three colonies of each mutant (STM-3Δ and STM-Δ*invA*) were cultivated, stocked in 25% of glycerol at −80°C, and checked by PCR and sequencing (Beckman Coulter).

### Eukaryotic cells and culture conditions

Epithelial cells from different species were tested: human colon adenocarcinoma HT-29 (ECACC), human hepatocyte carcinoma Hep G2 (ECACC), human cervical carcinoma cell line HeLa (ATCC CRM-CCL-2), minipig male ileum IPI-2I (ECACC), chicken hepatocellular carcinoma LMH (ATCC), African green monkey kidney Ma-104 (ATCC), human colon adenocarcinoma Caco-2 (ATCC), new-born piglet intestinal IPEC-1 (Gonzalez-Vallina et al., 1996) and murine hepatocyte AML-12 (ATCC). Three endothelial cell lines were also used: human brain microvascular endothelial cells HBMEC [kindly donated by Dr. C. Kieda (Centre de Biophysique Moléculaire, CNRS, Orléans, France)], human proliferating umbilical vein endothelial cells, HUVEC-p (PromoCell) and chicken aortic endothelial cells chAEC (Lion et al., 2017). Fibroblasts from different animal origins were also included: mouse primary kidney cloned, termed finite cell line RS-F1 (Velge et al., 1995), chicken fibroblasts DF1 (ATCC) and minipig male ileum finite cell line I31 (Kaeffer et al., 1993). Cells were routinely grown in 75 cm^2^ plastic tissue culture flasks at 37°C under 5% CO_2_ in the different recommended cell culture media without antimicrobial compounds.

### Adhesion and invasion assays

Gentamicin protection assays were performed as described previously (Roche et al., 2005). Briefly, the different steps of cell infection were analyzed with cells grown on 24-well tissue culture plates (Falcon) for 5 days to obtain subconfluent monolayers. Each experiment used two plates: one for the TCA step and the second for the invasion step. Cell monolayers were incubated in culture medium without antibiotics for 24 h and then infected for 1.5 h at 37°C with 10^7^ CFU in 300 μL in medium without serum (multiplicity of infection) MOI = 10. For TCA assays, the cell monolayers were gently washed six times with phosphate-buffered saline (PBS) (pH 7.3) and then disrupted with 1 mL cold distilled water (4°C). Viable bacteria (intra- and extracellular) were counted after plating serial dilutions on TSA. The other plates were washed with appropriate medium and incubated in culture medium containing 100 μg of gentamicin per mL. After 1.5 h at 37°C, cells were washed with PBS and lysed with 1 ml cold distilled water (4°C). Viable intracellular bacteria were assessed by serial dilutions plated on TSA (Tryptic Soy Agar—Difco). Results were expressed as the mean ± SEM of the number of intracellular bacteria for 10^7^ CFU. Experiments were performed in duplicate and repeated at least three times for each strain.

### Immunofluorescence

AML-12 cells were seeded in 24-well plates with 12 mm diameter glass coverslips at 10^5^ cells per well, 24 h before infection. Bacteria expressing pGG2-DsRed were grown in TSB with the required antibiotic for 7–8 h at 37°C with shaking, diluted 1:100 in fresh TSB, and incubated overnight at 37°C without shaking. Bacteria were diluted 1:5 and absorbance was measured. Then bacteria were added to the cells at MOI = 100 in medium without fetal calf serum. The infection was carried out for 1.5 h at 37°C in 5% CO_2._ Cells were washed four times with growth medium followed by twice with PBS and prepared for immunostaining.

Cells grown on coverslips were fixed with 3% formaldehyde (pH 7.4) in PBS at room temperature for 10 min. Fixed cells were washed three times in PBS, extracellular bacteria were stained first with rabbit primary antibody (*Salmonella* Typhimurium Antibody, BIOSS Inc.) and then with secondary antibody (Donkey anti-Rabbit Alexa 488) diluted in PBS and incubated for 1 h at room temperature with three intermediary washes. Cells were permeabilized with 0.1% saponin in PBS and the cytoskeleton was stained using phalloidin Alexa 647 at room temperature. Cell nuclei were stained with Dapi and coverslips were mounted on glass slides in Fluormount mounting medium. Cells were observed under a SP8 confocal laser-scanning microscope equipped with a 100× oil immersion objective (Leica). Images of 1,024 × 1,024 pixels were acquired using LaserX software (Leica). Sections 0.28 μm thick (38 per image) were assembled into Z stacks using Las AF lite 2.6.3 build 8173 software (Leica). **Figure 2** shows one section representative of the Z stacks.

### Scanning-electron microscopy

AML-12 cells were seeded in 24-well plates with 12 mm diameter glass coverslips at 10^6^ cells per well 48 h before infection, and then infected with STM or STM-3Δ at MOI = 100 for 15, 30, 45, 60, 90, 120, or 150 min. Cells were washed in PBS and fixed by incubation for 24 h in 4% paraformaldehyde, 1% glutaraldehyde in 0.1 M phosphate buffer (pH 7.2). Samples were then washed in PBS and post-fixed by incubation with 2% osmium tetroxide for 1 h. Cells were then fully dehydrated in a graded series of ethanol solutions and dried in hexamethyldisilazane (HMDS, Sigma). Finally, samples were coated with 40 Å platinum, using a GATAN PECS 682 apparatus (Pleasanton, CA), before observation under a Zeiss Ultra plus FEG-SEM scanning-electron microscope (Oberkochen, Germany).

### Translocation assay

A β-lactamase (TEM-1) translocation assay of T3SS-1 effector proteins SopD was performed (Charpentier and Oswald, 2004). Briefly, *S*. Typhimurium14028 and *invA* mutant carrying SopD::TEM-1 fusions or controls without fusion were incubated for 1.5 h with AML-12 cells at MOI = 100 in the presence of 1 mM probenecid (Sigma). Following infection, cells were loaded with CCF4-AM β-lactamase substrate (LiveBLAzer FRET-B/G Loading Kit, Invitrogen) for 2 h and visualized for green and blue fluorescence using confocal microscopy. Translocation was detected with fluorescence microscopy (SP8 confocal microscope Leica) using fluorescent CCF4/AM. Green fluorescence indicates CCF4-AM loading and the presence of blue cells is evidence of TEM-effector fusion translocation.

### Macropinocytosis and endocytosis assays

Invasion assays on an AML-12 cell line were performed in the presence of different inhibitors. AML-12 cells were pre-treated with drugs or their mock: amiloride (Sigma) at 1 mM for 30 min in culture medium, chlorpromazine CPZ (Sigma) at 10 μg/mL for 1 h in culture medium, monodansylcadaverine MDC (Sigma) at 300 μM for 1 h in DMSO, filipin (Sigma) at 5 μg/mL for 30 min in ethanol or genistein (Sigma) at 200 μM, for 30 min in DMSO. The dilution-effect of each drug was also performed. Viability of the bacteria was checked in the presence of all the dilutions of the drugs used. Bacteria (MOI = 10) diluted in the presence of the different drugs were deposited on cells for 1.5 h. Gentamicin was added for 1.5 h (using the same procedure as for the cell invasion assays). The number of internalized bacteria was determined and expressed in relation to values obtained for cells treated with control-diluted reagent arbitrarily set at 100%. Data are presented as the mean ± SEM. in duplicate and were repeated at least twice for each strain.

### Statistical analyses

Asymptotic two-sample Fisher-Pitman permutation tests (One-Way-Test) were performed with the software R, package Rcmdr version 2.3.1 (2016-10-25). Significance was ^****^ at *p* < 0.0001, ^***^p < 0.001, ^**^p < 0.01, ^*^p < 0.05 (http://www.r-project.org, http://socserv.socsci.mcmaster.ca/jfox/Misc/Rcmdr/).

## Results

### The *Salmonella* Typhimurium 14028 strain deleted for the three known invasion factors remains invasive in several eukaryotic cell lines

**Figure 1:**
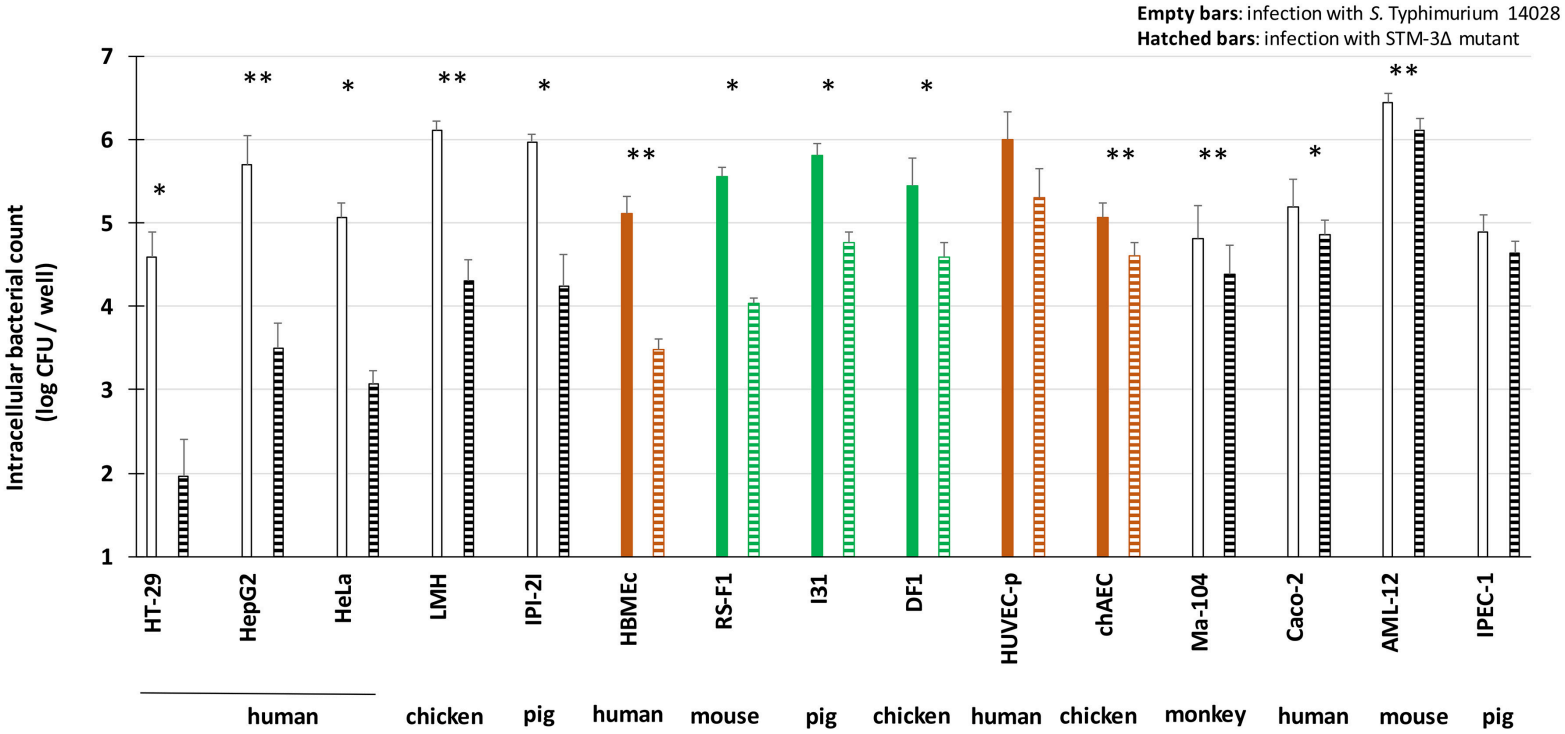
*S*. Typhimurium 14028 inactivated for the three known invasion factors remains invasive for several eukaryotic cell lines. Invasion abilities of *S*. Typhimurium 14028 and the STM-3Δ mutant were compared using gentamicin protection assays performed on different cell lines: epithelial cell lines (white bars), fibroblasts (green bars) and endothelial cell lines (brown bars). Bacteria (MOI = 10) were deposited on cells for 1.5 h followed by the addition of gentamicin (100 μg/mL) for 1.5 h. Empty bars represent the number of intracellular wild-type bacteria. Hatched bars represent the number of intracellular mutant bacteria. The results correspond to the mean ± SEM of at least three independent experiments performed in duplicate and expressed in log CFU/well. Cell lines were classified according to their dependence or not of T3SS-1 for invasion. Statistical analyses, using asymptomatic two-sample Fisher-Pitman permutation tests, were performed on intracellular bacterial counts between *S*. Typhimurium 14028 and STM-3Δ mutant for each cell type. Significance was ^*^P < 0.05 and ^**^ *P* < 0.01.

Both *S*. Typhimurium 14028 and STM-3Δ strains were investigated with gentamicin protection assays conducted on different cell lines. Fibroblasts, epithelial and endothelial cells from human, chicken, mouse, pig and monkey origins were tested. Primary fibroblasts (mouse primary kidney cloned, termed finite cell line RS-F1, minipig male ileum finite cell line I31) were also added. The numbers of total cell-associated (TCA) wild-type bacteria and its mutant, which correspond to surface adherent and intracellular bacteria, were not significantly different (data not shown). In the gentamicin protection assay, the cell lines were classified according to their dependence or not of T3SS-1 for invasion (Figure 1). For the majority of the cell lines, the mutant had an invasion defect, highlighting the T3SS-1 dependent invasion process (i.e., from HT-29 to RS-F1). For other cell lines (i.e., chAEC, Caco-2, Ma104, AML-12, and IPEC-1), the wild-type and the triple mutant had an invasion rate between 4.4 and 6.5 log CFU/well, and with the mutant's invasion rate being only 0.5 log CFU/well less than that of the wild-type strain. This result thus demonstrated that *Salmonella* remained able to enter some cells despite the absence of the three currently known invasion factors. However, no relation could be established according to the origin of the cells, as these results were obtained with four epithelial cell lines of different origins (Caco-2, Ma104, AML-12, and IPEC-1) and one endothelial cell line (chAEC). Moreover, no clear differences were observed between the primary cells and the cell lines. *E. coli* invasion was also checked in order to demonstrate that the uptake process observed by some cell types does not result from intrinsic ability to ingest bacteria independent of any virulence factors. Setting the uptake of the *S*. Typhimurium 14028 at 100%, the uptake of the *E.coli* MC1061 were 0.58, 0.63, 0.11, 2.01, 0.22, 0.38, respectively for AML-12, DF1, Ma104, HeLa, Huvec-p, and IPEC-1 cell lines.

### Significant T3SS-1 independent invasion is also observed after a brief contact between bacteria and cells

**Figure 2:**
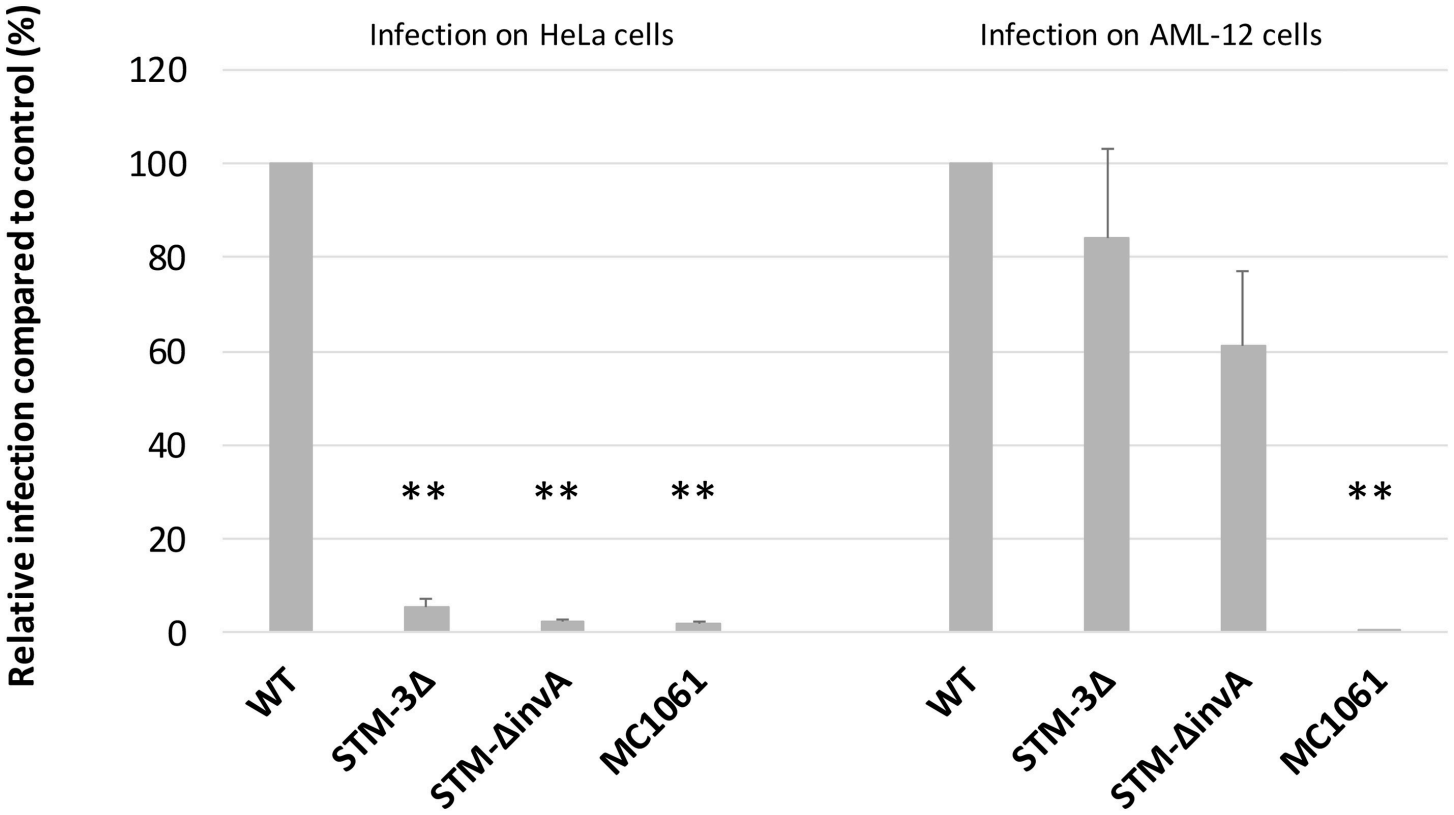
Significant T3SS-1 independent invasion is also observed even after a short infection time. Gentamicin protection assays were performed on HeLa and AML-12 cells. Bacteria (MOI = 10) were deposited on cells for 15 min followed by the addition of gentamicin (100 μg/mL) for 1.5 h. Experiments were performed three times in duplicate and expressed as a percentage of relative infection. Data were analyzed using asymptotic two-sample Fisher-Pitman permutation tests. Significance was ^**^P < 0.01. Statistical analyses also showed no significantly differences between the invasion rates of the STM-3Δ and the STM-Δ*invA* mutants (*p* = 0.1969 in the HeLa cells vs. *p* = 0.3514 in the AML-12 cells).

T3SS-1 is known to mediate cell invasion as early as 15 min after bacteria/cell contact. To assess whether T3SS-1 independent invasion can also be seen early after bacteria/cell contact, we performed a gentamicin protection assay on HeLa (a T3SS-1 dependent cell line) and AML-12 cells (a T3SS-1 independent cell line) with an infection time of 15 min instead of exposure for 1.5 h in 2.1. As a control, an *invA* mutant (T3SS-1 deficient) and a non-invasive *E. coli* (MC1061) strain were used. As expected, for HeLa cells, the *Salmonella* triple mutant and the *invA* mutant entered cells 19 and 40 times less than the wild-type strain, respectively. For AML-12 cells, the *Salmonella* triple mutant and the *invA* mutant entered cells 1.2 and 1.6 times less than the wild-type strain, respectively (Figure 2). These results showed that the T3SS-1-independent invasion of *Salmonella* into AML-12 cells occurs even after a short contact time, known to be sufficient for T3SS-1 invasion (Steele-Mortimer et al., 2002). Additionally, these data demonstrated that the entry of *Salmonella* into AML-12 cells was specific, since an *E. coli* strain entered this cell line at only a very low level.

### Microscopy reveals the uptake of the *S*. Typhimurium 14028 strain inactivated for the three known invasion factors by AML-12 cells

**Figure 3:**
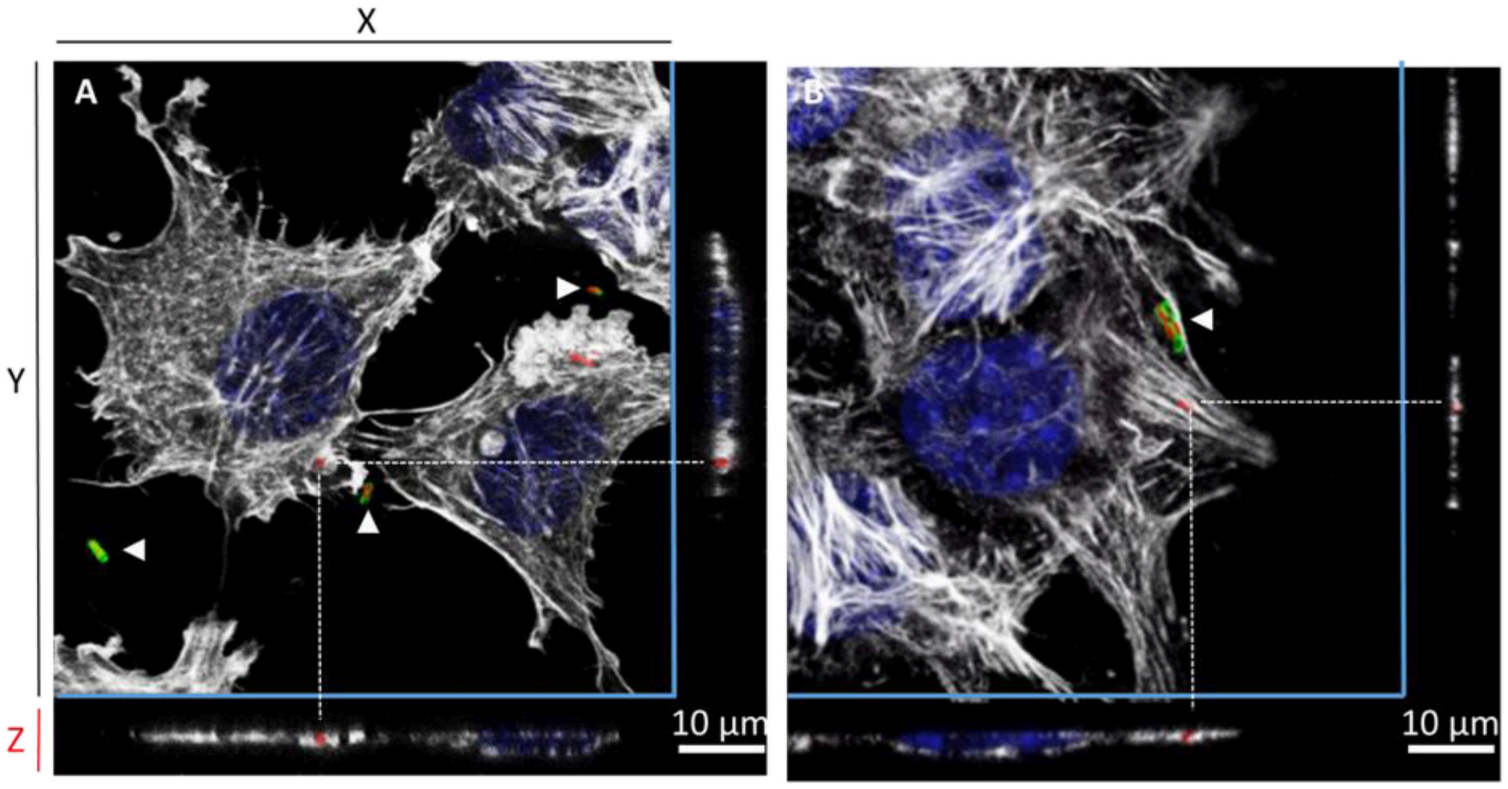
Confocal microscopy reveals the intracellular location of *S*. Typhimurium inactivated for the three known invasion factors in AML-12 cells. One projection of Z-stack sections, obtained by confocal microscopy (Leica SP8), of AML-12 cells infected for 1.5 h at 37°C (MOI = 100) with either the *S*. Typhimurium 14028 **(A)** or the STM-3Δ mutant **(B)** that expressed DsRed and were then processed by immunofluorescence. Intracellular bacteria were red due to DsRed expression or red and green (white arrowheads) when extracellular due to DsRed expression staining with a *Salmonella* O-antigen antibody revealed with goat anti-rabbit Alexa 488 (green) before permeabilization. Cell nuclei (DAPI) are shown in blue and actin in white (Phalloidin Alexa 647). Dotted lines indicate the position of intracellular bacteria in the Z-stack. Scale bar: 10 μm.

**Figure 4:**
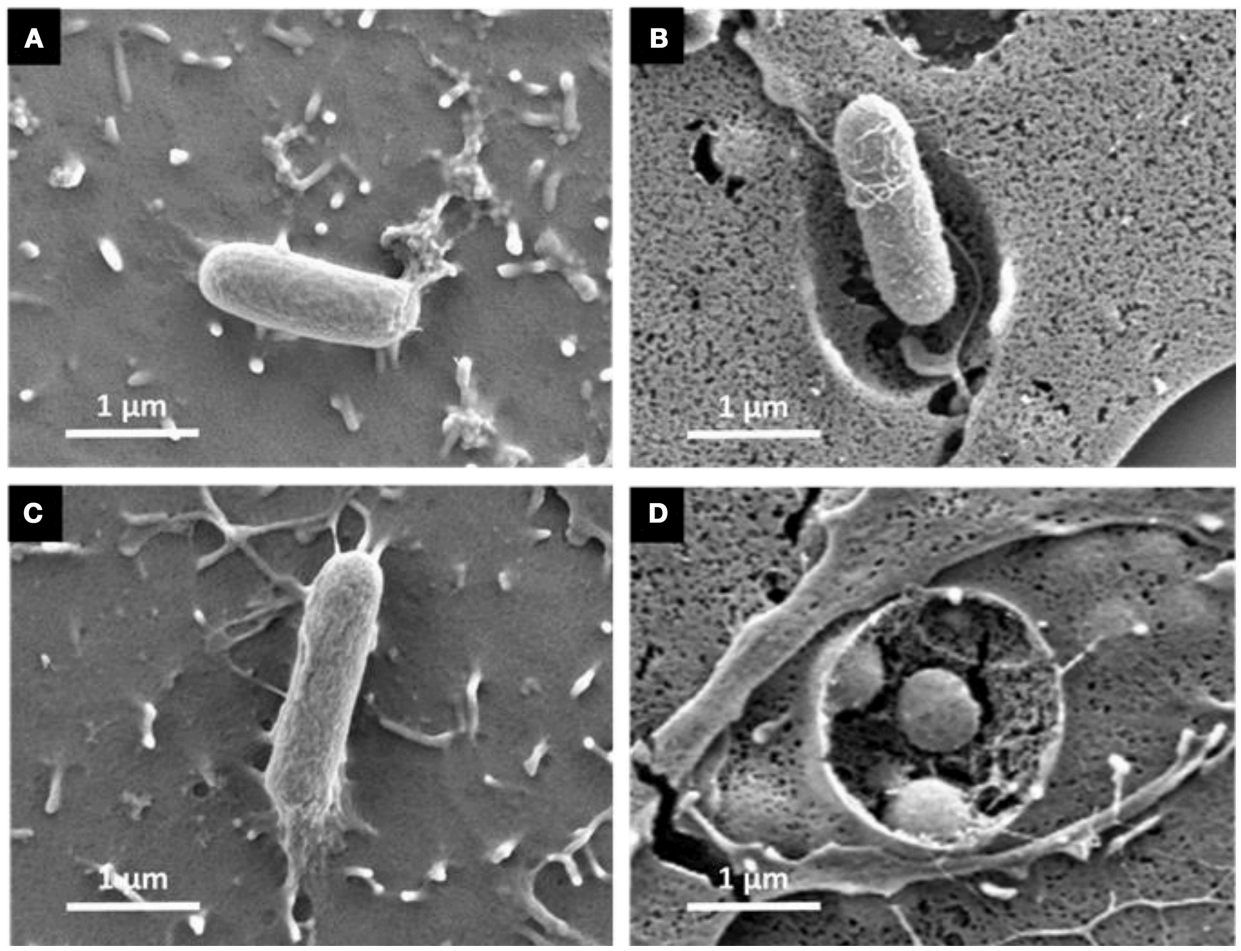
Scanning electron microscopy reveals weak cell surface rearrangements assuming a zipper-like mechanism for both the *S*. Typhimurium wild-type and its triple mutant. Cells seeded on glass coverslips at 70% of confluence were infected with either the *S*. Typhimurium 14028 **(A,B)** or the STM-3Δ mutant **(C,D)** at MOI = 100 then washed in PBS and processed for scanning-electron microscopy. Cells were then prepared for scanning electron microscopy. We selected representative observations of AML-12 cell surface rearrangements induced by *S*. Typhimurium 14028 and STM-3Δ infection. They showed filipodia contact with *Salmonella* (**A**−60 min and **C**−90 min) with weak membrane invagination surrounding *Salmonella* and engulfment (**B**−150 min and **D**−150 min). Bars: 1 μm.

The gentamicin protection assays revealed that the triple mutant entered AML-12 cells at a level close to that of the wild-type strain. Further experiments using confocal microscopy were thus carried out to confirm the intracellular localization of the wild-type and the STM-3Δ mutant. After verifying that no differences could be observed in the invasion rates between bacteria carrying or not pGG2-DsRed expressing DsRed (*S*. Typhimurium and STM-3Δ) (6.17/6.11 log CFU for the wild-type and 6.17/5.95 log CFU for the STM-3Δ), 70% confluent monolayers were infected for confocal microscope imaging. Intracellular *S*. Typhimurium expressing DsRed were visualized only red. Extracellular bacteria (adherent and free) labeled with a rabbit antibody directed against *Salmonella* and revealed with a green secondary antibody, were observed in red and green. Intracellular bacteria (in red) were observed and Z-stack acquisitions showed that they were embedded in white actin cocoons revealed by phalloidin staining (Figure 3). The recruitment of actin may reflect different levels of cell membrane rearrangement. As extensive or low cell rearrangement leads to trigger- or a zipper-like entry mechanism, respectively and as confocal imaging did not enable identification of the process involved, we next examined the morphological alterations occurring in AML-12 cells during bacterial uptake under a scanning-electron microscope (Figure 4). AML-12 cells tended to produce filipodia that came into contact with bacteria. Weak rearrangements were observed with cell invagination or bacteria wrapping, as already described in cells deficient in WAVE-complex function (Hänisch et al., 2010). These observations correspond to a zipper-like entry mechanism. Surprisingly, we did not observe any prominent membrane ruffles, the hallmark of the trigger mechanism, either with the wild-type bacteria (Figures 4A,B) or less surprising with the STM-3Δ mutant (Figures 4C,D). To ensure that these results were not time dependent, similar observations were conducted at different post-infection times (15, 30, 45, 60, 90, 120, and 150 min) (data not shown).

### Despite a zipper-like invasion, the T3SS-1 apparatus of the *S*. Typhimurium 14028 strain is functional in AML-12 cells

**Figure 5:**
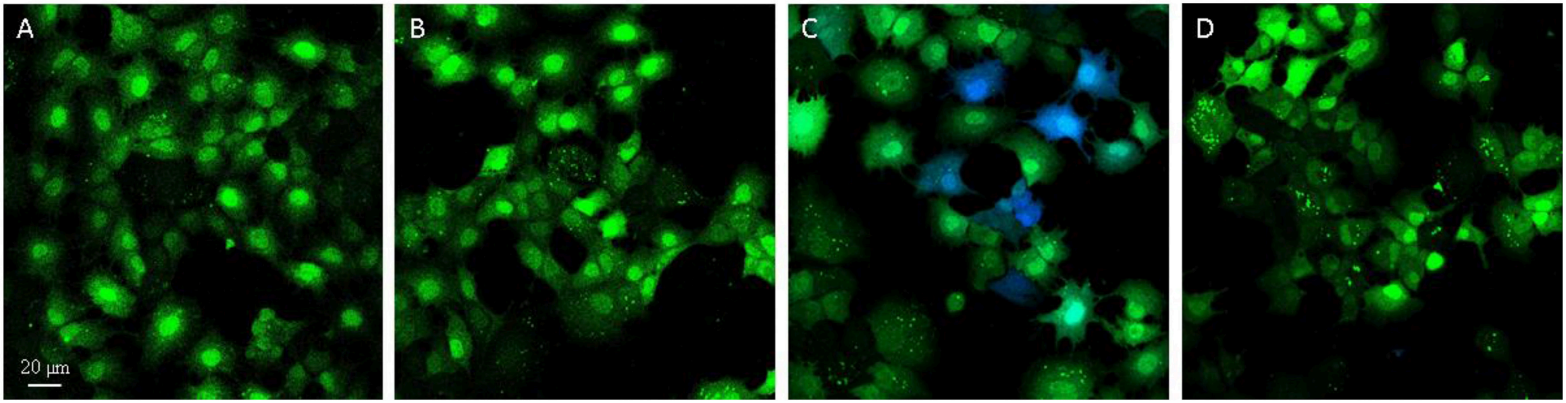
The T3SS-1 apparatus of the *S*. Typhimurium 14028 strain is functional in AML-12 cells. *S*. Typhimurium 14028 or Δ*invA* expressing SopD effector-TEM-1 β-lactamase were used to infect AML-12 cells for 1.5 h. Following infection, cells were loaded with CCF4-AM for 2 h and visualized for green or blue fluorescence through microscopy. Green fluorescence indicates that CCF4-AM was loaded and the presence of blue cells due to cleaved CCF4-AM revealed translocation. AML-12 cells were non-infected **(A)**, or infected with *S*. Typhimurium 14028 carrying the empty vector pCX340 **(B)**, *S*. Typhimurium 14028 expressing SopD effector-TEM-1 in pCX340 **(C)** and STM-Δ*invA* expressing SopD effector-TEM-1 in pCX340 **(D)**.

As a zipper-like invasion was observed with the wild-type strain, we investigated whether this strain was able to translocate T3SS-1 effectors into the host cell cytosol and thus whether its T3SS-1 apparatus was functional in AML-12 cells. For that purpose, we performed a fluorescence-based translocation assay using a fusion protein of the SopD effector with mature TEM-1 β-lactamase and fluorescent β-lactamase substrate CCF4-AM. The emission of blue fluorescence by AML-12 cells previously loaded with the substrate reveals cleavage of the CCF4-AM substrate by β-lactamase and therefore translocation of SopD into these cells. Uncleaved CCF4-AM emits green fluorescence. Blue cells were observed when AML-12 cells were infected with the wild-type strain carrying pCX340-sopD, the plasmid encoding SopD-TEM-1, demonstrating that *S*. Typhimurium 14028 was able to translocate the fusion protein into the host cell cytosol (Figure 5). In contrast, cells infected with the same strain carrying the empty vector pCX340 only emitted green fluorescence, indicating that conversion of the fluorescence of CCF4-AM (blue cells) was only due to the presence of the fusion protein in the host cell. This translocation was T3SS-1-dependent since the Δ*invA* mutant, which is unable to assemble a functional T3SS-1 and thus to inject T3SS-1 effectors, did not induce blue cells even when carrying the pCX340-sopD plasmid. These data demonstrate that *S*. Typhimurium 14028 was able to translocate at least the SopD T3SS-1 effector into the AML-12 cytosol indicating that the T3SS-1 was functional during the interaction of *S*. Typhimurium 14028 with AML-12 cells.

### Both clathrin- and non-clathrin-dependent pathways are used by a *S*. Typhimurium mutant inactivated for the three known invasion factors

Previous results showed that *S*. Typhimurium 14028 had a functional T3SS-1. However, no trigger invasion images were observed using scanning-electron microscopy.

To determine the pathways involved in the entry of the *S*. Typhimurium 14028 or the STM-3Δ, we tested chemical inhibitors that are commonly used to target macropinocytosis [amiloride (Koivusalo et al., 2010)], clathrin-mediated endocytosis [monodansylcadaverine, MDC Schlegel et al., 1982 and chlorpromazine, CPZ Wang et al., 1993], and the clathrin-independent, but lipid-raft-dependent route (filipin and genistein; Rejman et al., 2005).

**Figure 6:**
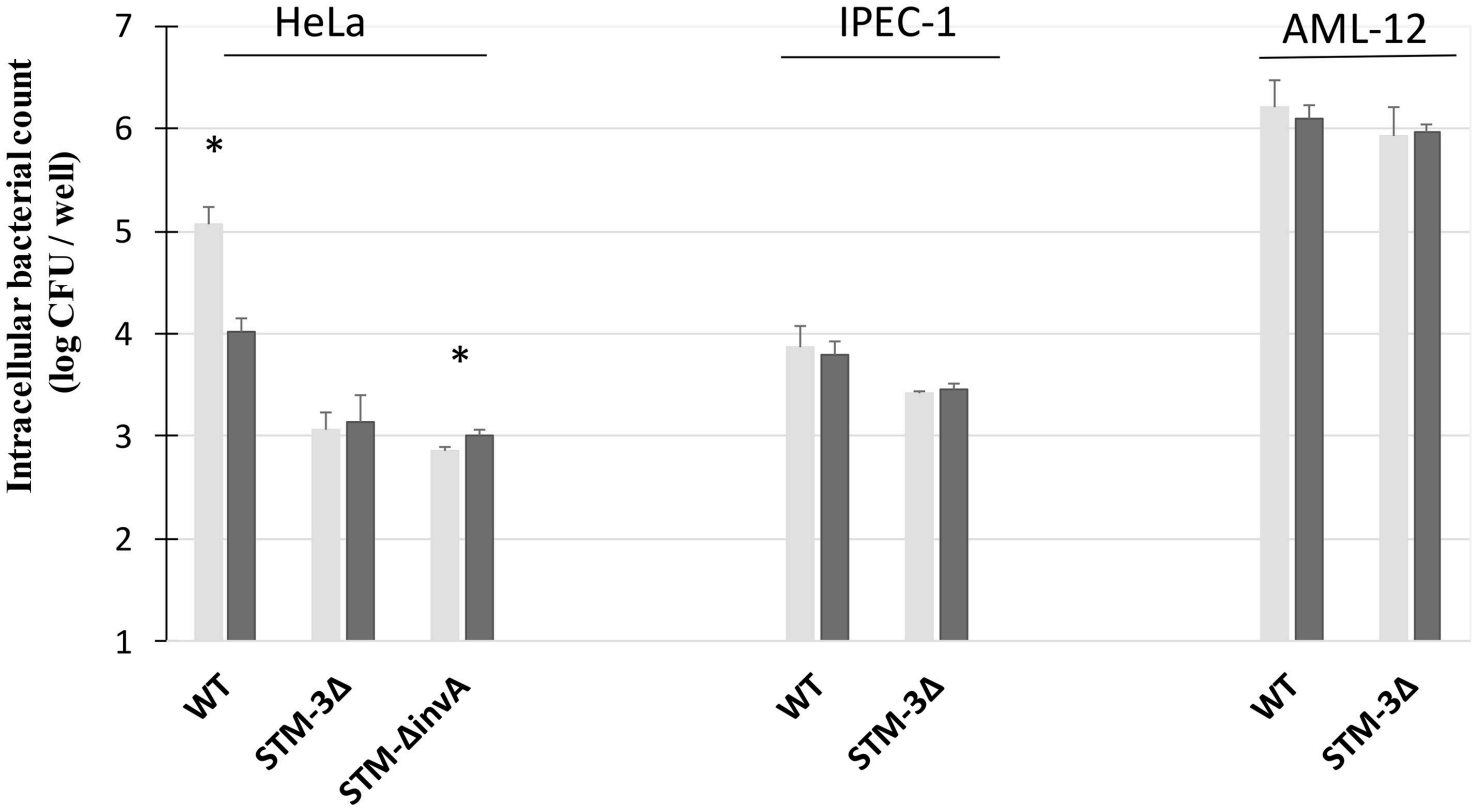
Amiloride affected the invasion of *S*. Typhimurium 14028 strain in HeLa cells T3SS-1 dependent-invasion. Invasion abilities of the *S*. Typhimurium 14028 and STM-3Δ mutant were compared using gentamicin protection assays performed on HeLa cells, AML-12 cells and IPEC-1 cells in the presence or not of amiloride. On HeLa monolayers, the single mutant 1invA was added as a control. Cells were pre-treated with amiloride (1 mM) (black bars) or the culture medium (gray bars) for 30 min. Bacteria (MOI = 10) were deposited on cells for 1.5 h followed by the addition of gentamicin (100 μg/mL) for 1.5 h. The numbers of internalized bacteria were determined. Data are the mean ± SEM in duplicate and repeated at least twice for each strain. Statistical analyses, using asymptomatic two-sample Fisher-Pitman permutation tests, were performed on intracellular bacterial counts between the infection in presence of culture medium (gray) or amiloride (black). Significance was *P < 0.05.

Previous studies showed that *S*. Typhimurium was internalized through macropinocytosis (Alpuche-Aranda et al., 1994; Rosales-Reyes et al., 2012) or that bacteria were associated with macropinocytosic vacuoles (Garcia-Del Portillo and Finlay, 1994). The effect of amiloride was tested on HeLa cells, a T3SS-1 dependent-invasion cell line (Figure 6). A factor of invasion was calculated in presence of amiloride or not. In accordance with previous papers, we observed that amiloride inhibited the entry of the wild-type bacteria. Indeed the wild-type bacteria entered 11.70 times better in absence of amiloride. By contrast, the entries of the single mutant Δ*invA* and the STM-3Δ were not altered in absence or presence of amiloride (factor 0.79, 0.72, respectively). These results reflect a T3SS-1 dependent-invasion process in HeLa cells. On the other hand, with AML-12 cells, amiloride did not affect the entry of the bacteria tested, and there were no significant differences between the wild-type and the triple mutant (factor 0.96, 1.00, respectively). To evaluate whether this phenomenon was specific to the cell line or to the invasion process, we tested amiloride on the IPEC-1 cell line, mediating another potential T3SS-1-, Rck-, PagN-independent-invasion process. Similarly, amiloride treatment had no effect on the entry of the wild-type or the STM-3Δ mutant (factor 1.22, 0.91, respectively) suggesting that the invasion process in these two cellular models was not related to macropinocytosis.

**Figure 7:**
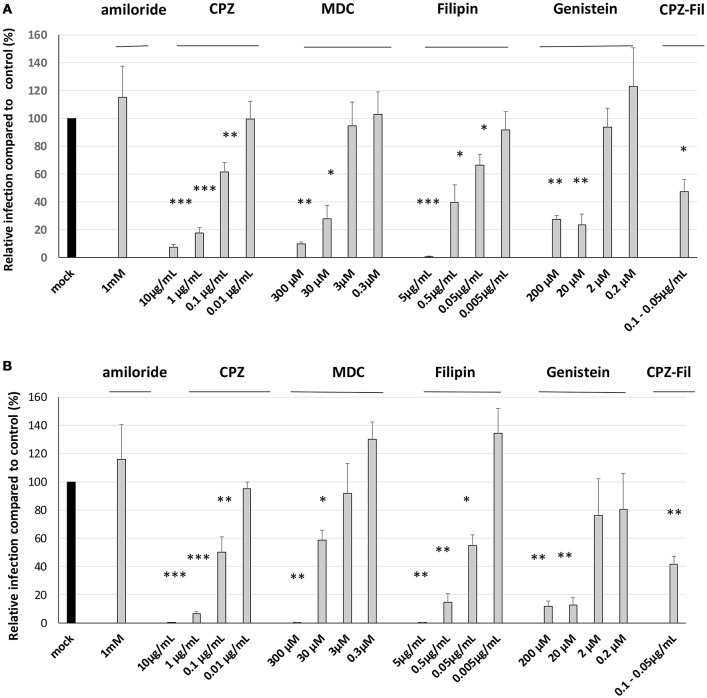
Both clathrin- and non-clathrin-dependent pathways are used by the *S*. Typhimurium 14028 strain and its triple mutant during interaction with AML-12 cells. Invasion abilities of the *S*. Typhimurium 14028 and STM-3Δ mutant were compared using gentamicin protection assays performed on AML-12 cells in the presence of different drugs. AML-12 cells were pre-treated with inhibitors at the indicated concentrations or with the appropriate solvent used for the dilution of the drugs for 30 min with amiloride (culture medium), filipin (ethanol) and genistein (DMSO), for 1 h with CPZ (culture medium) and MDC (DMSO). Bacteria (MOI = 10) were deposited on cells for 1.5 h followed by the addition of gentamicin (100 μg/mL) for 1.5 h. The dilution-effect of each drug was performed and viability of the bacteria was checked in the presence of all dilutions of the drugs used. The numbers of internalized bacteria were determined and expressed relative to values obtained for cells treated with control-diluted reagent arbitrarily set at 100%. Data are the mean ± SEM in duplicate and repeated at least twice for each strain. Data were analyzed using asymptotic two-sample Fisher-Pitman permutation tests. Significance was ^***^p < 0.001, ^**^p < 0.01, ^*^p < 0.05. **(A)**. represents the results obtained for the wild-type bacteria. **(B)**. represents the results obtained for the STM-3Δ.

For all the chemical compounds whatever the concentrations tested, the integrity of the cell monolayers was not affected. Further analyses showed that all drugs affecting the clathrin- and the non-clathrin-dependent pathways had an inhibitory and dose-dependent effect on *S*. Typhimurium 14028 and STM-3Δ invasion (Figures 7A,B). These findings combined with the fact that we could totally inhibit invasion, suggest that both pathways had a significant role in the entry of the wild-type strain and its isogenic mutant. As the drugs decreased the entry-rates of the bacteria to a similar extent, we decided to associate two different drugs, CPZ and filipin at a dose inhibiting 50% of the entry to demonstrate a cumulative or a redundant pathway. A cumulative effect would favor the existence of two distinct pathways. As no cumulative effect was observed, our result suggests that CPZ and filipin target the same internalization pathway. Moreover, when we compared the invasion rates of the wild-type bacteria and its mutant, no significant differences were obtained in their responses to the different drugs (Table 2) suggesting that both strains used the same entry process.

**Table 2:** Statistical analysis comparing the invasion rates of the wild-type strain and the STM-3Δ mutant, in the presence of drugs according to asymptotic two-sample Fisher-Pitman permutation tests (One-Way-Test) with the software R, package Rcmdr version 2.3.1 (2016-10-25).

	**CPZ (*p*-values)**	**MDC (*p*-values)**	**Filipin (*p*-values)**	**Genistein (*p*-values)**	**CPZ-filipin (*p*-values)**
Pure	0.006573^**^	0.01267^*^	0.3795	0.03427^*^	
Dilution 1/10	0.02669^*^	0.05497	0.1263	0.05884	
Dilution 1/100	0.3697	0.9171	0.293	0.5316	0.5712
Dilution 1/1,000	0.7431	0.2107	0.7901	0.2694	

Significance was at

** *p < 0.01*,

* *p < 0.05 (http://www.r-project.org, http://socserv.socsci.mcmaster.ca/jfox/Misc/Rcmdr/)*.

## Discussion

*Salmonella* Typhimurium invasion, and particularly the role of the T3SS-1, has been extensively characterized in cultured epithelial cells. This invasion process involves a subset of T3SS-1 effectors (SipA, SipC, SopB, SopE, SopE2) acting in concert to induce massive localized rearrangements of actin at the plasma membrane level, and also to activate signaling pathways resulting in membrane ruffling and macropinocytosis at the site of *Salmonella*-epithelial cell contact (Mcghie et al., 2009; Aiastui et al., 2010; Dunn and Valdivia, 2010). This entry process refers to a trigger entry mechanism. Moreover, Rosselin et al. demonstrated that Rck-coated beads and *E. coli* expressing Rck were able to induce a weak membrane rearrangement named the zipper mechanism in different cell lines (Rosselin et al., 2010). Consequently, a new paradigm has emerged based on the multiplicity of *Salmonella* entry mechanisms (Velge et al., 2012).

Our work provides some arguments supporting this new paradigm. A *Salmonella* triple mutant inactivated for the three known invasion factors, i.e., the T3SS-1 and the Rck and PagN invasins, is able to invade several cell lines at levels close to the wild-type strain, highlighting that uncharacterized invasion factors remain to be identified. However, this is not related to the cell origin (human, mouse, chicken, pig, and monkey) or the cell type (e.g., primary, epithelial, or endothelial cell lines). Our results also demonstrated that *S*. Typhimurium is able to invade some epithelial cells, according to a zipper-like mechanism, despite a functional T3SS-1. This observation is reinforced by the fact that macropinocytosis seemed not to be essential, as amiloride did not affect the invasion of two epithelial cell lines.

We also showed that the STM-3Δ mutant used the same entry pathway. In our model, the wild-type as well as the STM-3Δ mutant entered through a pathway involving both clathrin- and non-clathrin-dependent pathways. Veiga et al. demonstrated that bacteria injecting effectors into the host cells through a T3SS entered independently of a clathrin-dependent endocytic machinery, in contrast to the process observed for the entry of zippering pathogens (Veiga et al., 2007). However, this finding is not clear cut. First, their work was performed on a T3SS-1 dependent cell model and thus, depending on the cell used, either T3SS-1 dependent or independent, different entry pathways could be involved. In line with this, Cossart et al. showed that *Listeria*, which enters through a zipper mechanism, used both clathrin- and non-clathrin-dependent pathways (Cossart and Helenius, 2014). In the present study, we observed that the highest dose of chlorpromazine or filipin tested almost abolished *Salmonella* invasion to close to 100% suggesting that one endocytosis process was not compensated by another. These observations might indicate shared actors between the two endocytosis pathways or a partial specificity of the drugs used.

Our results showing that the wild-type strain did not use its T3SS-1 to enter cells despite a functional apparatus, demonstrate that the T3SS-1 is not required to infect some host cells. To date, most cell lines that have been used to study *Salmonella* invasion, (such as HeLa or HT-29 cells) are T3SS-1 dependent (Radtke et al., 2010). This could explain why invasion factors other than the T3SS-1 have not been identified easily. Recently however, other studies have shown that some mutants with their invasion altered, remained virulent *in vitro* and *in vivo*. For example, a mutant strain lacking the T3SS-1 effectors SopE and SopE2 was still able to invade fibroblast-like COS-7 cells in the absence of membrane ruffling (Stender et al., 2000). Similarly, a *S*. Enteritidis strain deleted for *invA*, not expressing Rck or PagN, was still able to invade fibroblast, epithelial and endothelial cells significantly using a zipper-like mechanism (Rosselin et al., 2011). Moreover, a *S*. Typhimurium ΔSPI-1 mutant entered rat and mouse fibroblasts (Aiastui et al., 2010), and *S*. Typhimurium mutants lacking the *invA* and *sipB* genes could infect human brain microvascular endothelial cells (Van Sorge et al., 2011).

In conclusion, *Salmonella* invasion of non-phagocytic cells is more complex than previously thought. Why the *S*. Typhimurium wild-type did not use its T3SS-1 to enter some cell lines is of fundamental importance and raises the question of the mechanisms that govern the initial steps of the invasion processes. How does the intracellular behavior of the bacteria change according to the entry factor used? Are the different entry processes involved in tissue tropism, host specificity and/ or disease outcome? Is there a synergy between the different entry mechanisms? The identification of the new invasion factors and the subsequent characterization of their entry mechanisms will undoubtedly help us to answer these questions.

## Author contributions

SR, SH, IV-P, and PV contributed to the conception and design of this study. SR, SH, SS, SG, and JT performed the experiments. SR and SH analyzed the data and wrote the manuscript. IV-P and PV discussed the results and improved the manuscript.

### Conflict of interest statement

The authors declare that the research was conducted in the absence of any commercial or financial relationships that could be construed as a potential conflict of interest. The reviewer BA and handling Editor declared their shared affiliation.
